# Comparison of Femoral Neck System Versus Cannulated Cancellous Screws for the Fixation of Femoral Neck Fracture in Young Adults: A Systematic Review and Meta-Analysis

**DOI:** 10.7759/cureus.32011

**Published:** 2022-11-29

**Authors:** Govind K Gupta, Arpita Rai, Subhankar Mandal, Sudha Rani, Shubhendu Shekhar, Subhajit Halder, Pancham Prasad, Amit Kumar, Zeya Ul Haque

**Affiliations:** 1 Orthopedics, Rajendra Institute of Medical Sciences, Ranchi, IND; 2 Oral Medicine and Radiology, Dental College, Rajendra Institute of Medical Sciences, Ranchi, IND; 3 Anatomy, Sheikh Bikhari Medical College, Hazaribagh, IND; 4 Laboratory Medicine, Rajendra Institute of Medical Sciences, Ranchi, IND

**Keywords:** meta-analysis, union time, harris hip score (hhs), cannulated cancellous screw (cc screw), femoral neck system (fns)

## Abstract

A femoral neck fracture is a very common injury in the elderly. However, its incidence is low among young adults, usually presenting as an emergency. In young adults, implant choice is one of the important factors. This systematic review aims to analyze the femoral neck system (FNS) versus cannulated cancellous (CC) screw for the fixation of femoral neck fractures in young adults through well-defined objectives. A comprehensive search from the electronic database (PubMed, Google Scholar, Web of Science, Cochrane Library) was conducted from the beginning till February 18, 2022. The data regarding study type, authors, year of publication, country, union time, Harris hip score, intraoperative blood loss, operating time, neck shortening, and hospital stay were extracted from the selected articles and analyzed using RevMan 5.4.1 software. For continuous data, e.g., healing time, intraoperative blood loss, operation time, Harris hip score, neck shortening, and hospital stay, the mean difference (MD), either weighted mean difference (WMD) or standardized mean difference (SMD), with a 95% confidence interval (CI) was recorded. A p-value less than 0.05 was taken as statistically significant. The Newcastle Ottawa scale was used for the risk of bias assessment. Six retrospective cohort studies including 427 patients were selected for the meta-analysis. There was significantly less healing time (WMD= -1.10, 95% CI: -1.73 to -0.47), shorter operation duration (WMD=7.70, 95% CI: -0.06 to 15.46), and better Harris hip score (WMD=4.79, 95% CI: 2.12-7.46) in the FNS than CC screw fixation method. However, intraoperative blood loss was significantly less in the CC screw system (WMD=21.27, 95% CI: 8.20-34.35). There was no significant difference between the two approaches in-hospital stay duration and femoral neck shortening. This can be concluded that FNS is better than CC screw fixation for treating neck of femur fractures in adults on the outcome basis of union time, less operation time, and better Harris hip score (HHS) with significant heterogeneity.

## Introduction and background

The femoral neck fracture is a very common injury in the orthopedic field [1]. It may occur in young patients with high-energy trauma such as falls from height or high-energy road traffic accidents. It accounts for only 3% of hip fractures in young patients [2]. In young patients, a femoral neck fracture is an emergency that has to be treated as soon as possible. As the delayed treatment may increase the chance of avascular necrosis. In young adults, the surgical intervention aims to preserve the biological head of the femur, avoid avascular necrosis or neck shortening, and help in early bone healing. Therefore, anatomical reduction and stable fixation are essential [3,4].

In young adults, implant choice has an important role in the success of the treatment. The usual treatment of choice is either closed reduction internal fixation (CRIF) or open reduction internal fixation (ORIF) with cannulated cancellous (CC) screw or dynamic hip screw (DHS), but there is a high risk of osteonecrosis, neck shortening, or non-union [4]. Pauwels type I and Pauwels II fractures are most commonly managed with three CC screws in an inverted triangle fashion. In the case of Pauwels type III fracture, basicervical neck and severely comminuted fracture treated with DHS is the preferred treatment. DHS gives better mechanical stability in such cases [3,4]. Due to the weak resistance power of the CC screw against vertical shear and torsion, a new minimally invasive technique named FNS provides stronger compression and angular stability. FNS was developed for dynamic fixation for the femoral neck fracture. It has a small side plate attached to the femoral shaft. The system consists of mainly three parts: anti-rotation (AR) screw, bolt, and side plate. The side plate provides angular stability, and the AR screw and bolt provide rotational stability [5].

The current systematic review and meta-analysis was carried out to compare two techniques, i.e., FNS versus CC screw, and to conclude the optimal surgical procedure for patients with a femoral neck fracture.

## Review

Materials and methods

This systematic review and meta-analysis analyzed the femoral neck system (FNS) and CC screw, which provides better results in terms of healing time and HHS for fixation of femoral neck fracture in young adults. The systematic review protocol was registered on PROSPERO (Registration number CRD42022313011) and was carried out according to the PRISMA guidelines and MOOSE checklist. The study population included all young patients (18-50 years) who suffered from femoral neck fractures and were surgically treated by CRIF or ORIF with CC screw or FNS. The initial displacement was reported according to the Garden classification. The primary outcome for analysis was union time, and HHS. The secondary outcomes were intraoperative blood loss (in ml), operation time (in minutes), hospital stays (in days), and neck shortening (in mm).

Documentation Sources and Search Strategies

We searched the articles from databases, e.g., PubMed, Google Scholar, Web of Science, and Cochrane Library, which were published from their inception till February 18, 2022. There was no language barrier. To handle the articles in languages other than English, we used translator services to read the article. We have searched the keywords in internet search engines such as Google to find out any relevant studies. We also searched all articles from the references of the most relatable articles. Our search keywords were (FNS OR Femoral neck system) AND (Cannulated cancellous screw or CC Screw) AND (Neck of femur fracture). Two authors (G.K.G. and S.M.) independently searched all related articles and their attribution in two phases, firstly reviewing the titles and abstracts thoroughly and then reading the full manuscripts for searching the important parameters.

Study Selection Criteria

We included six studies in our systematic review and meta-analysis. All the studies available were retrospective in nature. Case reports, case series, review articles, abstract publications, and conference presentations were excluded from the present systematic review. Studies with patients treated with dynamic hip screws or any other implant were excluded.

Data Extraction

Data were extracted by two investigators (G.K.G. and S.M.) independently by using a predesigned data extraction form. Any conflict between two authors was solved through discussion with a third author (S.S.) to conduct independent data extraction followed by discussion. The following data were extracted from each study: study type, authors, year of publication, country, union time, HHS, intraoperative blood loss, operating time, neck shortening, and hospital stay.

Assessment of Study Quality

The Newcastle Ottawa scale (NOS) was used to assess the bias of those studies independently. In this scale, a study can be awarded a maximum of one star for each numbered item within the selection and outcome categories. A maximum of two stars can be given for comparability.

Statistical Analysis

Review Manager (RevMan) software version 5.4.1 (Nordic Cochrane Centre, Cochrane Collaboration, Denmark) was used to conduct the meta-analysis. For continuous data such as union time, intraoperative blood loss, operation time, HHS, neck shortening, and hospital stay, the mean difference (MD), either weighted mean difference (WMD) or standardized mean difference (SMD) with a 95% confidence interval (CI), was recorded. A p-value less than 0.05 was taken as statistically significant.

Results

Search Results and Study Characteristics

**Figure 1:**
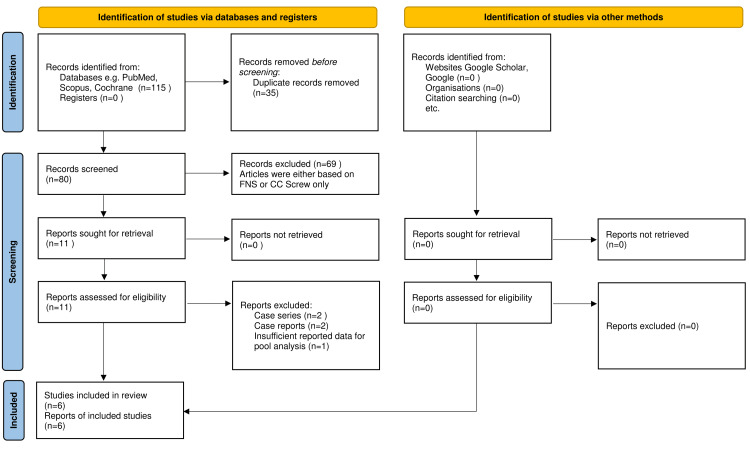
PRISMA Flow Diagram FNS, femoral neck system; CC screw, cannulated cancellous screw

A total of 115 potentially eligible studies were identified using the keywords for searching PubMed, Google Scholar, Web of Science, and Cochrane databases (Figure 1). A total of 35 articles were excluded for duplicity, while 80 articles were screened for their title and abstract. After removing the articles that were either based on CC screw or only FNS, 11 full-text studies were finally taken. Of these, six retrospective studies including 427 patients were taken for systematic review and meta-analysis owing to insufficient data available for pooled analysis in other retrieved studies (Figure 1).

The initial displacement according to the Garden classification was documented in Table 1.

**Table 1:** Classification of the patients in the studies as per the Garden classification CC screw, cannulated cancellous screw; FNS, femoral neck system

	Hu et al., 2021 [5]		He et al., 2021 [6]		Yang et al., 2021 [7]		Tang et al., 2021 [8]		Yan et al., 2021 [9]	
Garden classification	CC screw	FNS	CC screw	FNS	CC screw	FNS	CC screw	FNS	CC screw	FNS
Type I	4	0	2	1	0	0	0	0	2	0
Type II	6	6	8	9	4	5	5	6	10	4
Type III	7	8	19	20	16	12	31	29	32	12
Type IV	7	6	5	5	11	11	9	12	14	8

The study characteristics of each study along with all outcome variables are shown in Table 2.

**Table 2:** Demographic characteristics and outcome variables of the included studies FNS, femoral neck system; CC screw, cannulated cancellous screw

Study ID	Country	Follow-up period	Procedure	Number of patients	Male	Female	Union time (in months)	Blood loss (in mL)	Duration of surgery (in minutes)	Harris hip score
Hu et al., 2021 [5]	China	12 months	FNS	20	12	8	3.53±0.9	69.45±50.47	79.75±26.35	85.9±5.98
			CC screw	24	14	10	4.14±1.01	23.71±28.31	64.55±18.56	81.92±8.34
He et al., 2021 [6]	China	12–24 months	FNS	33	18	15	3.22±.37	NA	49.94±14.46	90.42±4.82
			CC screw	36	22	14	3.27±.059	NA	56.11±12.48	88.44±5.91
Yang et al., 2021 [7]	China	14 months	FNS	28	17	11	3.6±.9	27.8±6.3	45.5	92.4±5.5
			CC screw	31	17	14	4.4±.9	17.3±6.2	70	88.4±6.2
Tang et al., 2021 [8]	China	14–24 months	FNS	47	34	13	2.97±.35	50.6±10.6	52.4±11	88.9±4.3
			CC screw	45	37	8	4±.39	47.3±9.3	42±11.9	84.4±3.2
Yan et al., 2021 [9]	China	6-18 months	FNS	24	38	10	7.14±1.46	74.83±42.73	88.38±37.6	90±2.3
			CC screw	58	20	14	12.74±5.55	79.17±50.17	54.92±6.97	80.3±5.2
Zhou et al., 2021 [10]	China	10–22 months	FNS	30	12	18	5.23±1.33	99.73±52.73	42.82±4.69	86.16±7.26
			CC screw	30	12	18	6.03±1.45	30.27±9.04	40.9±5.22	82.37±7.52

**Figure 2:**
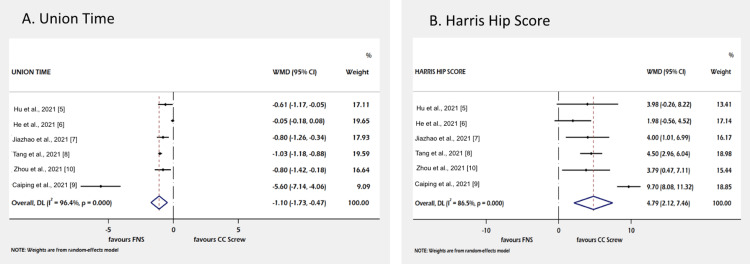
Forest plot of comparison of primary outcomes. Figure 2A shows union time and Figure 2B shows Harris hip score. Hu et al., 2021 [5]; He et al., 2021 [6]; Yang et al., 2021 [7]; Tang et al., 2021 [8]; Yan et al., 2021 [9]; Zhou et al., 2021 [10] CC screw, cannulated cancellous screw; CI, confidence interval; WMD, weighted mean difference; FNS, femoral neck system

The data from six retrospective studies reported the union time. It has been reported that the FNS group took less time for the union, which is statistically significant (WMD--1.10; 95% CI: -1.73 to -0.47) (Figure 2). The HHS is used for the assessment of functional outcomes in the two surgical groups by six studies, and pooled analysis showed a better HHS in the FNS group (WMD=4.79; 95% CI: 2.12 to 7.46) (Figure 2).

**Figure 3:**
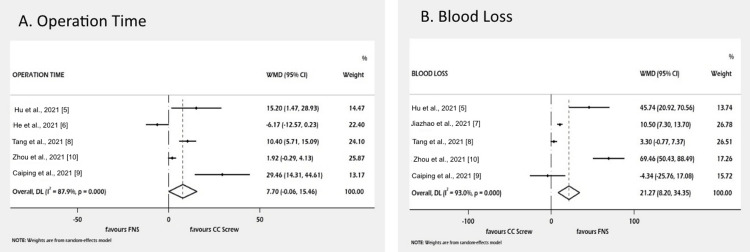
Forest plot of comparison of secondary outcomes. Figure 3A shows operation time and Figure 3B shows blood loss. Hu et al., 2021 [5]; He et al., 2021 [6]; Yang et al., 2021 [7]; Tang et al., 2021 [8]; Yan et al., 2021 [9]; Zhou et al., 2021 [10] CCs screw, cannulated cancellous screw; CI, confidence interval; FNS, femoral neck system; WMD, weighted mean difference

All six studies documented operative time, which is significantly higher in the CC screw group than the FNS group (WMD=7.70; 95% CI: -0.06 to 15.46). Similarly, five studies reported the amount of intraoperative blood loss, which is significantly less in the CC screw group (WMD=21.27; 95% CI: 8.20 to 34.35) (Figure 3).

Four observational studies showed mean hospital stay. No significant differences were found between the FNS group and the CC screw group (SMD=0.19; 95% CI: -0.04 to 0.42). Two studies documented femoral neck shortening, and there is no statistically significant difference (SMD=-0.27; 95% CI: -0.67 to 0.12).

Two studies documented non-union. Among them, one study showed 10% non-union in the FNS group and 12.5% non-union in the CC screw group. Another study showed 0% non-union in the FNS group and 5.6% non-union in the CC screw group. Only one study reported the number of fluoroscopies taken, which was much more in the CC screw group (18.33±33.82) in comparison with the FNS group (10.58±1.89).

The NOS was used for the assessment of the risk of bias in non-randomized studies (Table 3).

**Table 3:** Methodological quality for studies included (Newcastle Ottawa scale)

Study	Selection				Comparability	Outcome		
	Representativeness of the exposed cohort	Selection of the non-exposed cohort	Ascertainment of exposure	Demonstration that outcome of interest was not present at start of the study	Comparability of cohorts on the basis of the design or analysis	Assessment of outcome	Was follow-up long enough for outcomes to occur	Adequacy of follow-up of cohorts
Hu et al., 2021 [5]	*	*	*	*	*	*	*	*
He et al., 2021 [6]	*	*	*	*	*	*	*	*
Yang et al., 2021 [7]	*	*	*	*	*	*	*	*
Tang et al., 2021 [8]	*	*	*	*	*	*	*	*
Yan et al., 2021 [9]	*	*	*	*	*	*	*	*
Zhou et al., 2021 [10]	*	*	*	*	-	*	*	*

Discussion

This systematic review and meta-analysis was focused on the comparison of different types of outcomes with the FNS versus CC screw in femoral neck fractures in young adults.

The femoral neck fracture in a young adult is still a challenge to treat in clinical practice because there is a high chance of post-operative complications such as avascular necrosis, non-union, and neck shortening. Perfect anatomical reduction and strong stable internal fixation are the important factors to minimize the aforementioned complications [11]. The ideal surgical technique for femoral neck fracture fixation is still controversial. It should include some characteristics such as less surgical time, less intraoperative blood loss, less postoperative morbidity and mortality, a short duration of hospital stay, lesser effective cost, and early mobilization of the patient to improve the patient’s daily work life as well as reduce the complication related to being bedridden such as bed sore, pulmonary complication, and deep vein thrombosis. Stoffel et al. [12] compared the biomechanical property in Pauwels type III femoral neck fracture treated with FNS or CC screw or (dynamic hip screw) DHS in a cadaveric model. The study reported no significant differences between DHS versus proximal femoral nailing or FNS as per biomechanical properties, but both are superior to CC screw fixation. In our review, a statistically significant difference has been reported in union time between the two groups (WMD=-1.10; 95% CI: -1.73 to -0.47), which favors that the FNS group unites faster than the CC screw group. The assessed union time might be affected by the follow-up duration, but all included studies except one have reported similar follow-up duration for both groups. The follow-up duration ranges from 6 to 24 months. Our results coincide with the findings mentioned by Rajnish et al. in their study [13].

FNS provides a stable and strong fixation to reduce post-operative varus deformity. FNS also allows for controlled collapse/compression. CC screw applies pressure at the fracture site, which helps in bone healing. Compared to the FNS group, CC screws occupy less space in the neck and head area, which interferes less with the arteriovenous supply of the neck and head of the femur. Conventional triangular fashion placement of CC screws gives three-dimensional constructions, which can reduce the stress of femoral head rotation. But the position of the screws may be easily affected because it depends on some subjective or objective factors. These subjective factors may be the surgeon skill, fracture type, reduction technique. The objective factor may be the implant material. Therefore, its vertical shear and torsion resistance power is poor, which may cause the fracture site loosening and displacement [14,15].

CC screw fixation is a percutaneous procedure, and in the case of FNS fixation, 5- to 7-cm lateral incision is required near a greater trochanter, which may cause more soft tissue exposure [16]. According to our meta-analysis, there was significantly more blood loss in FNS than in CC screw fixation procedure (WMD=21.27; 95% CI: 8.20 to 34.35)]. The finding was similar to the previous review [13,17].

It is difficult to put three CC screws in a parallel inverted triangle fashion in an unstable displaced femoral neck fracture. Chen et al. documented a study of stable and unstable femoral neck fractures treated with CC screw and showed 4.5% non-union and 9.1% avascular necrosis [18]. In young adults, femoral neck fracture occurs due to high-energy trauma, which leads to extensive damage to the blood supply, and the correct surgical intervention itself jeopardizes the blood supply leading to avascular necrosis [19]. Due to weak resistance power against vertical shear and torsion, fixed-angle devices are used for better angular stability and compression. The FNS makes a 130-degree angle with the femur lateral plate with the bolt, which helps achieve static compression and fixation of the fracture site. FNS’s AR screw prevents the rotation of the femoral head, which reduces the chance of damage to the newly forming capillary. Therefore, FNS decreases the chance of non-union and avascular necrosis. The operative time is reported significantly less in the FNS group than in the CC screw group (WMD=7.70; 95% CI: -0.06 to 15.46).

Many studies showed that FNS has a better biomechanical property than CC screw fixation for the treatment of Pauwels type III fracture because it gives more structural and rotational stability. Xu et al. used FNS for femoral neck fracture and reported a satisfactory result. There was less chance of post-operative complications such as loosening of the implant or neck shortening [20]. Our meta-analysis reported that the HHS is significantly higher in the FNS group (WMD=4.79; 95% CI: 2.12-7.46) than in the CC screw group.

Fracture fixation is directly proportional to bone quality. Any patient with the features of osteoporosis showed excessive compression at the fracture site during the early healing process, which may cause absorption at the fracture site, which leads to femoral neck shortening [21,22].

In a developing country, cost-effectiveness is an important factor for patient compliance. FNS is much more expensive than CC screw.

The imported implant cost is much higher. The cost for one FNS from DePuy Synthes (Johnson & Johnson Medical Devices, New Brunswick, NJ, USA) is approximately Rs. 45,000. One CC screw costs approximately Rs. 6,000. One patient usually needs three CC screws; therefore, the cost will be approximately Rs. 18,000. The above implants are made up of titanium [23]. Stainless steel implants are cheaper as compared to titanium. Also, implants made by Indian companies are cheaper than other international companies. The cost for Made in India (such as Bonetech Medisys Pvt Ltd) is comparatively lesser [24]. One FNS costs approximately Rs. 27,000 and one CC screw costs approximately Rs. 1,500. So the cost of three CC screw implants required for a case is around Rs. 4,500. The material used in both implants is titanium. The above cost description may vary slightly as per location, and additional charges might be applied for the actual procedure of surgery.

In our systematic review and meta-analysis, we found that FNS is better than CC screw fixation for femoral neck fracture in a young adult because of better structural stability, strong sliding compression facility, and size of the FNS plate, which is suitable for Asian people and reduces the soft tissue irritation around the plate.

Limitations of the study

The present study has some limitations. The studies included in the meta-analysis were retrospective, and these are prone to various biases. The result reported in this review has marked heterogeneity. The cause of this heterogeneity might be the clinical differences, methodological differences such as issues with randomization, early termination of trials, dissimilar follow-up durations of the studies, and protocol followed by the studies. Therefore, the results of retrospective studies have to be interpreted with caution. All the included studies were from a single country, thereby limiting the widespread applicability of the findings. More studies can evaluate to choose the better fixation method effectively. Therefore, more studies with proper study design are required to evaluate and choose a better fixation method for effective and better functional outcomes of FNS and CC screw.

## Conclusions

The current study compared the two techniques, FNS and CC screw fixation, in the treatment of femoral neck fractures in young adults. The result revealed that there is a significant difference in union time, operation time, and HHS, which favors FNS over the CC screw fixation method. Only blood loss is less in the CC screw group. Significant heterogeneity was observed among the studies; therefore, the finding should be interpreted with caution. No significant differences were found between FNS and CC screw groups for the hospital stay and femoral neck shortening.
